# Biomechanical effect of neurologic dance training (NDT) for breast cancer survivors with chemotherapy-induced neuropathy: study protocol for a randomized controlled trail and preliminary baseline data

**DOI:** 10.21203/rs.3.rs-2988661/v1

**Published:** 2023-06-29

**Authors:** Kristen D Lantis, Patrick Schne, Courtney R. Bland, Jacqueline Wilder, Karen Hock, Nelson A. Glover, Madeleine E. Hackney, Maryam B. Lustberg, Lise Worthen-Chaudhari

**Affiliations:** College of Medicine, Department of Physical Medicine and Rehabilitation, The Ohio State University, Columbus, Ohio; College of Public Health, Division of Biostatistics, The Ohio State University, Columbus, Ohio; College of Public Health, Division of Biostatistics, The Ohio State University, Columbus, Ohio; College of Medicine, Department of Physical Medicine and Rehabilitation, The Ohio State University, Columbus, Ohio; Comprehensive Cancer Center, The Ohio State University, Columbus OH; George Mason University, Fairfax, VA; Department of Medicine, Division of Geriatrics and Gerontology, Emory University School of Medicine, Atlanta, GA; Atlanta VA Center for Visual and Neurocognitive Rehabilitation, Atlanta, GA; Yale Cancer Center, New Haven, CT, US; College of Medicine, Department of Physical Medicine and Rehabilitation, The Ohio State University, Columbus, Ohio

**Keywords:** chemotherapy-induced neuropathy, oncology, neurorehabilitation, music, dance, biomechanics

## Abstract

**Background:**

Breast cancer (BC) is among the most common forms of cancer experienced by women. Up to 80% of BC survivors treated with chemotherapy experience chemotherapy-induced neuropathy (CIN), which degrades motor control, sensory function, and quality of life. CIN symptoms include numbness, tingling, and/or burning sensations in the extremities; deficits in neuromotor control; and increased fall risk. Physical activity (PA) and music-based medicine (MBM) are promising avenues to address sensorimotor symptoms. Therefore, we propose that we can combine the effects of music- and PA-based medicine through Neurologic Dance Training (NDT) through partnered Adapted Tango (NDT-Tango). We will assess the intervention effect of NDT-Tango v. home exercise (HEX) intervention on biomechanically-measured variables. We hypothesize that 8 weeks of NDT-Tango practice will improve the dynamics of posture and gait more than 8 weeks of HEX.

**Methods:**

In a single-center, prospective, two-arm randomized controlled clinical trial, participants are randomly assigned (1:1 ratio) to the NDT-Tango experimental or the HEX active control intervention group. Primary endpoints are change from baseline to after intervention in posture and gait. Outcomes are collected at baseline, midpoint, post, 1mo follow up, and 6mo follow up. Secondary and tertiary outcomes include clinical and biomechanical tests of function and questionnaires used to compliment primary outcome measures. Linear mixed models will be used to model changes in postural, biomechanical, and PROs. The primary estimand will be the contrast representing the difference in mean change in outcome measure from baseline to week 8 between treatment groups.

**Discussion:**

The scientific premise of this study is that NDT-Tango stands to achieve more gains than PA practice alone through combining PA with MBM and social engagement. Our findings may lead to a safe non-pharmacologic intervention that improves CIN-related deficits.

**Trial Registration:**

This trial was first posted on 11/09/21 at ClinicalTrials.gov under the identifier NCT05114005.

## Background and rationale

Breast cancer (BC) is among the most common forms of cancer experienced by women, second only to skin cancer(1). Up to 80% of BC survivors treated with chemotherapy experience chemotherapy-induced neuropathy (CIN)(2, 3), a dose-limiting physiological effect of cancer treatment that degrades motor control(4, 5), sensory function(4), and quality of life(3). Neurologically, CIN involves axonal dieback as well as central deficits(6) with even one cycle of chemotherapy altering cortical thickness in the sensorimotor regions of the brain(7). Symptoms of CIN persist into survivorship as numbness, tingling, and/or burning sensations in the feet and hands(8); measurable functional deficits in neuromotor control(4, 9–15); and increased fall risk(16). Pharmacologic treatment of CIN has failed to improve sensorimotor symptoms or remediate motor function directly while creating additional negative effects(17) and interactions with key treatment agents(18). Despite the high prevalence and debilitating consequences of neurotoxic chemotherapy exposure, treatment options remain limited(8).

Physical activity (PA) is a leading candidate to treat CIN non-pharmacologically(19–24), purportedly through inducing axonal regeneration(30), central plasticity(48, 49), and other health benefits such as improved aerobic capacity(25) and reduced systemic inflammation(26). However, more work is needed to motivate habitual participation and optimize neurorecovery potential. Music-based medicine (MBM) is another promising avenue through which to address sensorimotor symptoms(27). Music triggers neurophenomena in humans and other species(28–31) that manifest functionally as auditory-motor entrainment(32), functional neuromotor stabilization(33, 34), and pain reduction(27, 35–37). We propose that we can combine the positive effects of music- and PA-based medicine through dance as neurologic training. Neurologic Dance Training (NDT) may optimize neurorecovery potential over and above MBM and PA, separately, by activating the neuropathic dynamic system(38) in the context of rhythmic auditory stimulation(33, 39), sensorimotor skill acquisition(40), creative engagement(41–43), and social engagement(44).

To test the hypothesis that NDT improves CIN symptoms more than PA, we propose to test the effect of NDT in the form of social dance versus (v.) PA in the form of home exercise training. Specifically, we will evaluate the effect of Adapted Argentine Tango practice (NDT-Tango), a light-moderate intensity social dance adapted for persons with mobility deficits(45, 46), on functional and patient-reported symptoms of chronic CIN among breast cancer (BC) survivors. Research by the multiple principal investigators (MPIs) established NDT-Tango as feasible for aging survivors (up to 82 years old (yo)) to engage in biweekly at a mean(SD) dose of 33(4) minutes (min)/session with high satisfaction and positive effects(44, 47). We hypothesize that NDT-Tango will improve function and sensation among BC survivors who demonstrate CIN with balance dysfunction.

## Objectives

The primary objective of this experiment is to assess the intervention effect of partnered NDT-Tango v. home exercise (HEX) intervention on biomechanically-measured functional variables that predict fall risk among BC survivors with CIN and postural control deficits. Secondary measures include functional and patient-reported outcomes (PROs) related to CIN symptom load and/or fall risk. Additionally, we will evaluate within-session effects of training on postural control and PROs. We hypothesize that 8 weeks (wks) of NDT-Tango practice (2x/week (wk); 15–30 min dose of movement-to-music/session) will improve the dynamics of posture (Aim1) and gait (Aim2) more than 8 wks of HEX (2x/wk; 30–60 min dose of PA/session) among BC survivors with CIN and demonstrated balance dysfunction.

## Methods

### Study design

In a single-center, prospective, two-arm randomized controlled clinical trial design, eligible participants who provide informed consent are randomly assigned to the NDT-Tango experimental or the HEX active control intervention group in a 1:1 ratio. The study primary endpoints are change from baseline to after 8 wks of intervention in biomechanically-measured variables of dynamic function that predict fall risk: specifically, change in the primary outcome measures of postural control variability(48–51) (Aim1) and coefficient of variability for stride speed (CVspeed)(47, 52) (Aim2). Table 1 presents the data collection schedule, formatted per the Standard Protocol Items: Recommendations for Interventions Trials (SPIRIT) recommendations(53–55). Figure (Fig) 1 depicts the overall study design as a flow diagram. As detailed in Table 1, outcomes are assessed at baseline (i.e., repeatedly over 2 wks prior to intervention), midpoint (i.e., after 4 wks of intervention), endpoint (i.e., after 8 wks of intervention), 1 month (mo) follow up (I.e., 1 mo after intervention completion), and weekly in the 6 months (mos) following intervention end. Secondary outcome measures include clinical and biomechanical tests of function that complement the primary outcome measures, patient-reported outcomes, and falls incidence. Tertiary outcome measures, depicted in Table 2, include within-session effects, satisfaction collected at session end, and surveys used to inform monitoring and shaping of interventions including: rating of perceived exertion (RPE; 6–20 scale) and Intrinsic Motivation Inventory (IMI). In addition, at the suggestion of participants, we repurposed the RPE to query mental exertion v. physical exertion and call this a Rating of Perceived Mental Exertion (RPME; 6–20 scale).

#### Repeated baseline schedule

Select measures, indicated in Table 1 with an *, are collected repeatedly prior to intervention to characterize within-subject variability (WSV) in CIN-related deficits and symptoms at baseline (Fig. 2). This is relevant for clinical decision-making because WSV represents the typical day-to-day means and variability per measure that clinicians can expect to see among patients with CIN. Knowledge of WSV per measure enables longitudinal tracking of maintenance, improvement, or decline in function for individual patients(48). Repeated baseline characterization of postural control is achieved through collection of the postural control dynamics during silence (QEC) test at the beginning of up to 5 visits, including the first screening test of postural control and 3–4 additional days. Within the current paper, we report results of this characterization of postural control variables known to predict future falls,(48) and results of our assessment regarding whether there is an effect of measuring postural dynamics repeatedly during this baseline period of testing. In addition to QEC, we collect postural control dynamics during music listening (QECm) and PROs at the beginning of baseline visits (Fig. 2). At the end of baseline visits we collect postural control and PROs again as well as rating of physical and mental exertion perceived during any activity involved in baseline testing (i.e., MiniBEST, 6mwt). To confirm the expected effect of no physical activity, we conduct at least one “resting” baseline test in which participants remain seated and engage in conversation with research staff for 20 minutes before re-testing QEC, QECm, and PROs (Fig. 2). This extensive repeated baseline design allows us to compare intervention activity effects relative to outcome assessment activity (i.e., 6mwt, MiniBEST) and no physical activity (i.e., 20 minutes of conversing with research staff).

### Study setting

The study is currently being conducted at The Ohio State University (OSU) and the research protocol has been approved by The OSU Institutional Review Board. Participants are recruited from the Stephanie Spielman Comprehensive Breast Center (SSCBC) oncology outpatient facility as well as from the greater central Ohio (OH) community. Consent is obtained in a quiet and private setting prior to research activity either electronically, via REDCap, or using a paper version which is stored securely in a locked cabinet within secure research-dedicated space at the host institution. Assessments (i.e., screening, repeated baseline, midpoint, post intervention, and 1 month follow up assessments) are performed in an outpatient care clinic setting or within the volunteer’s home environment, as preferred by the survivor. The Tango intervention is performed at the outpatient care clinic in a group setting of up to 5 survivors at a time with their invited partners. The HEX intervention is performed by the participant in their own home environment. Participants are compensated $20 per in-person assessment session that does not involve interventional instruction with an additional $20 compensated for sessions that require sensors to be applied to the skin (potential total of $180 for participation in all assessments).

### Eligibility criteria

Participants are eligible to join the study if they are > = 40 yo, diagnosed with BC (stage I-III), experiencing CIN (CIPN-20 sensorimotor score > 1), finished with taxane-based chemotherapy treatment for at least 3 months, able to understand and comply with directions associated with testing and study treatments, and if they demonstrate postural control measurements outside of normative values(48). Participants are excluded from the study if they meet any of the following exclusion criteria: preexisting vestibular disorders, history of motor deficits or neurological disease other than CIN-related, poorly controlled diabetes (hgA1 C ≥ 8), non-ambulatory or lower extremity amputation (assistive devices allowed), participating in physical therapy during the study, or contraindicated to participate in unsupervised activity due to other issue (e.g., herniated vertebral disc).

We expect the study population to be representative of the demographics of the BC survivorship population. The CDC reports that 99% of BC cases occur among women and only 1% among men. Therefore, we anticipate that those who participate in this study will be almost exclusively women. The CDC census reports the rate of new BC cases in 2019 per 100,000 women by race as: White/Non-Hispanic 132.5, Black/Non-Hispanic 128.4, American Indian & Alaska Native/Non-Hispanic 101.7, Asian & Pacific Islander/Non-Hispanic 104.5, and Hispanic 101.9(56).

### Recruitment and screening

The research opportunity is posted on public facing websites including ClinicalTrials.gov (NCT05114005), Research Match, and Study Search to allow volunteers to self-refer by calling a dedicated medical center phone line or emailing the secure address Tango@osumc.edu. Furthermore, advanced practice providers (APPs) refer eligible and interested clients to our research staff to complete screening. Research staff work on site within the SSCBC outpatient facility at least 2 days per week to follow up with volunteers and referring APPs. In addition, queries are run within electronic medical records (EMR) to identify potentially eligible clients of the OSU academic medical center system. Reports are generated using the criteria of a taxane-based chemotherapy plan being entered in the EMR and filtered by age (>/= 40 years) and prescribing physician. Through chart review, we identify the subset of clients who meet these study inclusion criteria: (a) CIN symptoms reported within their last clinic visit on record, (b) no vestibular or neurologic disorder other than CIN, (c) no uncontrolled diabetes (A1C < 8.0), and (d) not currently participating in physical therapy.

For those identified as eligible, we request approval from the treating oncologist to contact the client for recruitment purposes. Eligible clients who are approved for recruitment by their oncologist are contacted by phone to inquire whether CIN symptoms persist and, if so, to determine interest and availability in participating in this study. Those able and interested to participate are asked to schedule in-person screening of postural control, which involves attempting a short but challenging balance task reported to distinguish fallers from non-fallers by Maki et al (1994)(49). As previously reported(57), to perform this task volunteers stand quietly and bilaterally for 30 seconds (s) on a balance plate (Bertec Corp, Columbus, OH) with eyes closed eyes (QEC). Center of pressure (COP) variables of interest are calculated per Prieto(58) and Roerdink(59). Survivors are offered enrollment in the study if they demonstrate QEC postural control function that is outside of the estimated 70% confidence interval (CI)(48) of healthy, age-equivalent normative values in (1) COP ellipse area, (2) medial-lateral variability, (3) medial-lateral velocity or outside of the estimated 95% CI in terms of (4) COP complexity. Values corresponding to these inclusion thresholds are reported in Table 3.

### Adequacy of the potential participant pool

Approximately 1200 new BC patients are seen annually at the OSU SSCBC, 240 + of whom will receive taxane-based cytotoxic chemotherapy annually. We expect at least 50% of BC patients treated with cytotoxic chemotherapy to experience persistent CIN and measurable postural control deficits(48, 60) yielding an eligible patient pool that grows by approximately 120 BC survivors per year or 10 eligible individuals per month. Given the interest in non-pharmacologic options for survivorship treatment and lack of pharmacologic interventions to treat CIN(61), we anticipate that approximately 20% of eligible patients will be interested and consent to enroll in the study for a projected accrual rate of 2 new participants enrolled per month. In addition to this annual presentation of new individuals with BC, OSU houses an established survivorship program that serves thousands of BC survivors, including survivors not seen within our medical center, who have been living with chronic issues of survivorship such as CIN for years. Finally, these survivorship efforts are continually growing their reach by addressing and overcoming issues of access to survivorship programming.

Lastly, attending classes with an invited guest (partner) was found to affect engagement, by improving attendance of survivors(44). Therefore, we encourage participants to invite a partner to attend training sessions with them and, for those who prefer not to invite from their social circle, we provide partners from a pool of talented university students within the OSU Department of Dance and School of Health and Rehabilitation Sciences to activate social engagement for enrollees randomized to the Tango experimental intervention.

### Interventions

Both interventions – NDT-Tango and HEX – occur over an 8-week period at a frequency of 2x per week. No intervention session in either arm will last longer than 1.5 hrs. total.

#### Experimental Intervention

##### NDT-Tango.

The NDT-Tango intervention will consist of 16 Adapted Argentine Tango (Adapted Tango) sessions, adapted for neurorehabilitation by Co-I Hackney (45, 62), and taught by PI Worthen-Chaudhari. Per session, we aim to deliver a NDT dose (defined as skilled movement-to-music) of at least 10 minutes and no more than 35 minutes, with breaks for water and rest offered at least every 10 minutes. Recommendations for implementation of Adapted Tango as a neurologic intervention focus on prevention of falls, use of implicit learning techniques to convey skilled movement goals, the structure of class (warm-up, lesson, cooldown), scaffolding and shaping lessons over time to establish competency in fundamentals, modifications deemed necessary for specific deficits, and music selection(45, 62). Musical compositions (songs) used within this intervention are from the tango genre with a duration of 1.5 to 5 min. Participants may sit or rest as needed during or between songs. Instructors ensure appropriate activity wear choices (e.g., footwear, breast support). In addition, instructors ensure qualified volunteers are present to partner with participants. Instructors complete 16 hours of training with Co-I Hackney as well as a certification exam to become qualified to teach the Adapted Tango technique. Volunteers who will partner participants for this study complete Co-I Hackney’s 4 hour Balance Management program plus an additional 3 hours of training with PI Worthen-Chaudhari during which live instruction skills are evaluated by the PI before volunteers are cleared to partner participants.

The MPIs (Worthen-Chaudhari and Lustberg) previously demonstrated that an NDT-Tango dose of 32.9(3.9) minutes movement-to-music is feasible for cancer survivors with postural control deficits to participate in at a rate of 2x per wk. To optimize factors mediating intrinsic motivation, instruction aims to achieve high Enjoyment and Perceived Competence among participants with low Pressure/Tension(63–65) as measured by the Intrinsic Motivation Inventory (IMI). We administer the IMI every 2 wks to assess achievement of these instructional goals. To assure integrity of the training, Co-I Hackney assists MPIs Worthen-Chaudhari and Lustberg to monitor fidelity of the intervention. This subset of our team – representing 2 former professional dancers turned dance scientists and 1 oncology practitioner - monitor participant symptoms and training progression and troubleshoot any issues that arise through weekly virtual meetings.

##### Active Control Intervention: Home Exercise.

The active control intervention consists of an evidence-based, structured home exercise program (HEX) based on the 8 wk PA intervention for CIN described by Zimmer et al (2018)(23). This PA design was modified for home exercise delivery among BC survivors with CIN in collaboration with OSU-based leaders in physical therapy who specialize in BC oncology rehabilitation (e.g., co-author Hock). This program combines endurance, resistance, and sensorimotor training for 45–60min per session performed 2x per wk(23). Participants in the HEX group will be provided with a yoga mat and resistance bands with which to perform the interventional exercises. HEX participants will be prompted to report adverse events, symptoms, and barriers to participation 2x per week through the Research Electronic Database Capture (REDCap) MyCap application that is installed on participant smart phones. HEX instructional materials include (1) a manual with pictures and text descriptions per exercise that can be shared as a pdf document or paper binder and (2) short demonstration videos per exercise, viewable on a dedicated YouTube channel (i.e., @Tango_HomeExercise). HEX participants will be given the direct YouTube link and the channel will remain private, meaning only accessible by those with the link. Following completion of repeated baseline testing, research staff will work with HEX participants in a single 1:1 session to instruct in PA performance and modify exercises as needed in collaboration with the participant. Research staff with expertise in exercise instruction, including PI Worthen-Chaudhari who is certified through the American Council on Exercise (ACE) as a Medical Exercise Specialist (CMES), will determine what PA level HEX participants will start with: seated, beginning, moderate, or advanced. Research staff will contact HEX arm participants 2x per week to discuss potential required intervention shaping and modification as well as to offer social connection, a schedule designed to match social connection provided to participants of the experimental intervention group. If barriers to participation are identified, research staff will address by modifying the exercise form, challenge level, duration, and/or schedule of PA. For instance, participants who advance beyond the moderate challenge level of HEX exercise performance will be provided with the option of using a pair of 3-pound cuff weights to increase resistance challenge above what is provided by resistance bands. Participants who complete the 8-week HEX intervention will be offered participation in 16 Tango lessons after study completion.

#### Procedures/plans to ensure safety of interventions

##### Pain management:

It will be critical to address the possibility that pain may interfere with training sessions (66–69). While both music and movement have the potential to reduce cancer-related pain, and our prior work did not identify pain as a barrier to participation in the intervention, nevertheless, a structured plan is indicated for assessing and addressing the potential for pain in this population. Use of gabapentin, opiates, and/or duloxetine for neuropathic pain management will be allowed and addressed in statistical analysis as concurrent medications. We will monitor pain as we have previously (70), using question 6 from the Brief Pain Inventory, a visual analog scale (VAS) on which participants will rate how much pain they are in “right now” on a scale of 1 to 10 (10 being high). Pain ratings will be collected before and after each training session. Using a scheme we applied previously to guide intervention trials in neuropathic populations (71–73), if pain at the start of a session is more than 2 points above the start of the prior session, we will consider training to be contraindicated. If pain at the end of a session is more than 2 points above pain at the start of that session, we will require 2 days of rest before attempting the next training session and will not commence future training sessions until pain is within 2 points of the last training start. If pain remains elevated, or participation is not possible, for 4 sessions in a row then study participation will be terminated. Pain monitoring of NDT-Tango participants will occur at the time of each session and procedures will be carried out in-person by the PI via 1:1 discussion with the participant in a private setting. Pain monitoring of HEX participants will occur bi-weekly, via review of MyCap entries, and procedures will be carried out by research staff via phone contact.

###### Functional guarding

The Tango partner hold (aka “embrace” or “frame”) provides an opportunity to support participants in the upright position as well as to guard against falls during Tango movement practice. To train staff and volunteers in how to apply the Tango partner hold, and other techniques for best practices in functional guarding, Co-I Hackney conducts a 4hr standardized fall prevention (Balance Management) training program with each study personnel. This fall prevention training is in addition to Hackney’s 16hr certification program, with certifying exam, that prepares staff to teach Tango as a medically relevant activity for persons with neuropathology and is a standard offering from ACE.

###### Inclement weather

In the event of weather that creates unsafe driving conditions for planned in-person NDT-Tango intervention sessions, a video conference link (i.e., Zoom) will be sent to participants as an option through which to participate. Those who choose to participate will be instructed to position themselves in the corner of a room and/or near a stable surface (e.g., back of a stable chair that is not on wheels; countertop) such that the walls of the room and/or stable surface are available for support as needed. Participants will be instructed to aim the camera toward their position, enable video sharing, remain unmuted, and join computer audio. No partner hold will be used; instead, participants will face the video display and mirror movement demonstrated by instructors in their camera view. At least one research staff person will monitor participant feeds to ensure safety throughout the video conference period.

###### Seated activity option

There is a chance that functional impairments or deconditioning may create challenges with prolonged standing (i.e., more than 1 minute of standing at a time). Participants in both arms may stop to rest as desired. For the NDT-Tango arm, shorter compositions of 1.5 minutes or less will be used when participants are challenged with sever impairment or deconditioning. If we enroll a participant who cannot stand for longer than 1 minute at a time, then that participant will be encouraged to sit as needed and to continue HEX or NDT-Tango movement from the seated position.

###### Depression and anxiety monitoring

The psychosocial state of participants is relevant to this project in terms of recruitment, retention, and effect. Depression and anxiety tends to be higher among cancer survivors than in the general population(74); it is important to capture these aspects of patient experience. The Patient-Reported Outcome version of the **Common Terminology Criteria for Adverse Events v5 (PRO-CTCAE),** developed by NCI, is standard protocol in oncology to screen for signs of depression and anxiety associated with cancer treatment or progression(75). We use the CTCAE to screen for sadness and anxiety per visit and will monitor and compare descriptive statistics between groups. Increases in severity of sadness or anxiety of 2pts or greater between sessions are addressed by the MPIs. Monitoring of NDT-Tango participants occurs at the time of each session; the PI invites the participant to leave the group setting to discuss. Monitoring of HEX participants occurs bi-weekly; MPIs reach out to the participant via phone contact.

###### COVID management plan

COVID vaccination was publicly available by the time of recruitment start in Aug 2021. Per medical center policy, we strongly encouraged COVID vaccination and maintained masking among all participants and staff during in-person visits. While COVID vaccination status was not codified as an inclusion criteria, we held separate Tango group sessions for vaccinated and unvaccinated participants as requested.

### Outcome Measure Procedures and Analysis

#### Primary Outcome Measures

##### Postural dynamics (Aim1)

We measure postural dynamics as postural control, or sway. The primary outcome, postural control variability, is measured through biomechanical data collection during a challenging 30 second (s) balance task, that has been described previously(48, 57). Briefly, participants are instructed to stand quietly and bilaterally for 30 seconds with eyes closed (QEC) on a portable balance plate (Bertec Corporation, Columbus, OH), with feet set apart (5cm), and arms relaxed at their sides. To control for the effect of auditory stimulation(33), the test is performed in silence within a quiet environment. To control for known responses to vision obstruction, participants are asked to focus on a point approximately 8 ft in front of them at eye level prior to closing their eyes at the moment collection commenced(4, 44, 48, 57, 70) and in response to the researcher’s countdown of “3..2..1..close”. Biomechanical data describing postural sway are acquired using custom software written in LabVIEW at a sampling frequency of 1000Hz and consist of one channel of force data (Fz) and two channels of moment data (Mx, My) over time. From these data we calculate the center of pressure (COP) time series for the 30 second duration of QEC condition performance. We then calculate the primary outcome measures of COP variability in resultant and mediallateral planes (RMSr, RMSml) for this 30 second duration per Prieto(58).

##### Gait dynamics (Aim2)

We measure gait dynamics as gait variability(34, 52, 76) and stability(77–79) using data from inertial measurement units affixed to the foot, leg, thigh, pelvis, and/or lower cervical spine during the 6-min walk test (6mwt). The primary outcome measure for Aim2, CVspeed, will be calculated from acceleration data(80). Steady-state walking epochs are detected from gyroscopic data.

#### Secondary and Tertiary Outcome Measures

##### Postural dynamics:

Starting from the QEC COP time series data described *in postural dynamics (primary outcome measure),* we calculate additional linear(58) and non-linear(59) secondary outcome measures. In addition to linear primary outcome measures (1) RMSr and (2) RMSml we calculate linear secondary outcome measures (3) variability in the anterior-posterior plane (RMSap) (4) 95% ellipse area (CoPa), 5) path length (PL) and (6–8) mean velocity in resultant, medial-lateral, and anterior-posterior planes (COPvr, CoPvml, COPvap). We analyze one non-linear measure: sample entropy (SEI) of the resultant CoP position using the increment calculation method with constant values applied of m = 3 and r = 0.3 (59). Entropy of postural responses represents the automatic complexity(81) of neuromotor control that is available to an individual and has been found sensitive to disease states such as brain injury(82) and neurodegenerative disease(83–86) as well as to skill mastery(87) and attentional focus(59, 88, 89). Within this manuscript we report descriptive statistics for these primary and secondary outcomes describing postural control and for select clinical measures of function among BC survivors enrolled to date (n = 23). Within-subject variability at baseline as well as intervention effects will be reported in future publications.

##### Function:

We measure function using the following validated clinical tests, instrumenting some of them accomplish biomechanical quantification of the motion performed during the tests. All functional tests are performed in a clinical space or the participant’s home in an area that is distraction free The *Timed Up-and-Go test, or TUG,* is a timed test of a person’s ability to stand from a chair, walk 10 feet, turn around, and return to sitting(90) with shorter times indicating better functional balance. Presence of CIN has been associated with longer TUG times(11–14). To measure dual-task function, we use the TUG performed simultaneously with a cognitive task consisting of audibly counting backward by 3s or 4s *(TUGCog).* Blackwood and Rybicki (2021) reported that the TUGCog threshold of ≥ 11.32s identified BC survivors at risk of falling (sensitivity = 0.64 and specificity = 0.8)(13). To measure dynamic balance function we collect *Mini Balance Evaluation System Test (MiniBEST),* which evaluates sensory organization, anticipatory and reactive postural control, and dynamic gait indices(5) and was found to discriminate BC survivors from controls in at least 1 prior study(12). Finally, to measure physical function in terms of endurance and mobility we collect *6mwt,* in which shorter distances correlate with poorer function including among survivors with CIN(91). Meta-analysis of 6mwt results among adults with pathology or fear of falling indicate that 14.0–30.5m represents the minimum clinically-important difference (MCID) for improvement in this measure(92). The 6mwt is performed within an oncology clinic, in a quiet area without distractions. Participants walk in a loop with the following configuration: a straight distance of 65 feet (ft) minimum marked by tape on the floor that participants turn around to their left; participants are cued to walk straight between tape marks and to turn comfortably at the end of each straightaway at the fastest pace they feel they can maintain for 6 minutes, resting as needed in a standing position before continuing.

##### Gait dynamics

Measures of walking variability and stability have been found indicative of mild cognitive impairment(93), age(94), fall risk(52, 76, 95), and neuropathy(96, 97) regardless of age or type of cancer(98) including specifically in BC(10, 97, 99). Using a portable inertial measurement unit (IMU) system while participants perform the 6mwt(100), stride-to-stride measures of variability previously found sensitive to health status will be calculated from the straight, steady-state sections of the 6mwt loop pattern(47, 52, 76). Local dynamic stability(101–104) and orbital stability(10, 104, 105) will also be analyzed, as measures that have been shown sensitive to neuropathy and used previously by our team to measure intervention effect among BC survivors(106).

##### Co-contraction index (CCI)

is a ratio of the activity produced in agonist v. antagonist muscles of the leg that provides unique insight into neuropathy effects(107). We calculate this ratio from electromyographic (EMG) signals of the tibialis anterior, gastrocnemius, and soleus muscles. We calculate this muscle activity ratio during performance of select MiniBEST sensory organization tests as well as during QEC and QECm postural dynamics trials and the 6mwt.

##### Influence of music listening on postural dynamics:

We measure the influence of music listening on postural dynamics (QECm) per the protocol established by Ross et al.(33). After completing the QEC trial (and always in this order), the participant is asked to step off the balance plate and walk or march in place for a few steps before being asked to step back on the plate into the QEC posture while we begin playing the musical composition “La Cumparsita” at a volume that is deemed loud but not uncomfortable by the participant. Participants are instructed to look at a defined spot approximately 8 feet away, during approximately, and no less than, 10s of acclimating to auditory stimulus, before being cued to close their eyes for collection of the QECm trial. Auditory acclimation is accomplished in the following way: we instruct participants to listen to the music, focusing specifically on the periodic rhythm (i.e., “beat”). To facilitate beat perception, we provide external cueing (i.e., snapping fingers or clapping hands to the musical beat) for approximately 4s. We then extinguish external cueing over a 2s period, by quieting then ceasing the cueing sound. Then participants are allowed to acclimate to auditory stimuli, without external cueing, for an additional 4s before research staff verbalize the directions “3..2..1..close”, speaking one word per beat for a measure of 4 counts and start of postural control data recording as participants close their eyes. COP-based variables of interest are calculated as described for the QEC trial.

##### Patient-reported outcomes (PROs):

Patient self-report surveys are administered through The Ohio State University administered instance of the REDCap database for in-person visits and through the REDCap platform’s MyCap mobile application (MyCap) for data entry that participants perform remotely (e.g., HEX activity, fall tracking in 6 mo follow up). Answers are reviewed manually by authorized research collaborators, who prompt participants to complete or clarify answers as needed and document their responsibility for review from a list of authorized data reviewers. Within this study we collect the following PRO measures: *(i) European Organization for Research and Treatment of Cancer’s Quality of Life Questionnaire, Chemotherapy-induced Peripheral Neuropathy (CIPN-20):* is a validated 20-item patient reported questionnaire instrument for longitudinal evaluation of neuropathy symptoms induced by chemotherapy(108). While validated for a recall period of “during the past week” we use modified forms of the CIPN-20 to query items for the recall periods of “during the activity just performed” (at the end of sessions) and “right now” (at the beginning and end of sessions). *(ii.) The Brief Pain inventory (BPI)* is validated for ecological momentary assessment (EMA) of immediate and retrospective (prior 24 hours) self-reported pain and relevant functional capacity in our target population(109). *(Hi.) The Brief Fatigue inventory (BFI)* is validated for EMA of immediate and retrospective (prior 24 hours) self-reported fatigue and relevant functional capacity in our target population. *(iv.) Ecological momentary assessment (EMA) of immediate symptomatology.* Using the Patient-Reported Outcome version of the Common Terminology Criteria for Adverse Events (PRO-CTCAE), cancer-related symptoms such as balance problems, nausea or vomiting, dizziness, sensitivity to light or noise, feeling like “in a fog”, confusion, sadness, and anxiety can be queried for the recall period of the past 7 days(108, 109). This study requires query of symptomatology in the immediate moment, or “right now”: we capture these symptoms in this timeframe using an EMA tool that prompts for self-report of symptom severity “right now” called the Sports Concussion Assessment Tool (SCAT) symptom inventory(110). The SCAT was created for and validated among individuals with mild traumatic brain injury, however, the SCAT symptom inventory queries the desired PRO-CTCAE items (i.e., 39–56) in the time frame of “right now” using a 7-point Likert scale (0 = no incidence; 6 = high severity), which affords greater sensitivity than the PRO-CTCAE 4 point Likert scale (1 = no incidence; 4 = high severity), and has been validated for use among individuals with known cognitive deficits. Therefore, we use the SCAT symptoms inventory in lieu of the PRO-CTCAE to query the desired subset of the PRO-CTCAE symptoms with regard to how participants feel “right now” with sensitivity and in a format appropriate for individuals who might have cognitive deficits associated with chemotherapy exposure. *(v.) Activity tracking:* Participants are asked if they have participated in the following activities: physical therapy, occupational therapy, fitness activity, other therapeutic or fitness activity. If any category of activity is checked as having occurred then participants are prompted to estimate the amount of time spent doing the activity since we last saw them (hours, minutes). (*vi*) *Falls tracking:* Falling is defined as an unexpected loss of balance in which an individual comes to rest at a position lower than before the unexpected event(111). We elicit self-report of falls and loss of balance using the question “How many times have you fallen or felt like you lost your balance since we last saw you?” Responses are typed by the participant and reviewed for incidence of falls, incidence of loss of balance, and details offered about either incidence. *(vii.) Barriers to participation:* Participants are asked “To help us understand barriers to participation, if you missed a session, please indicate the reason why you were unable to attend (e.g., schedule conflict, transportation, didn’t feel up to it, forgot).” *(viii.) Satisfaction with intervention* is measured after each class using a 7pt Likert scale and prompt for feedback about what did/did not work per class. Feedback is used to improve future sessions. *(ix.) The Intrinsic Motivation inventory (IMI)* was developed from the perspective of Self Determination Theory to assess 7 dimensions of experience: Interest/Enjoyment, Perceived Competence, Effort/Importance, Pressure/Tension, and Choice(63–65, 112–114). We administer the 9 item short form of the IMI monthly to optimize instruction around low Pressure/Tension and high Interest/Enjoyment(63, 64) as well as to explore relationships between adherence, motor effects, and IMI dimensions including perceived benefit (i.e., Effort/Importance).

### Power Calculations

We targeted an effect size in postural control variability of 1.22 (Cohen’s *d)* based on preliminary data from five cancer survivors with postural control deficits at baseline who participated in Tango practice. Enrolling 26 participants per group (52 total), allows for a drop-out rate of up to 45% to yield full follow-up on 14 participants per arm and 85% power to detect the target effect size at the 5% significance level. This drop-out rate is conservative, corresponding to criteria that we used previously to assess an intervention’s feasibility(44, 115). Regarding gait variability (i.e., CVspeed), primary outcome for Aim2, our preliminary data show an effect size of 0.73 (Cohen’s *d)* from seven survivors, not screened for CIN or postural control deficits, who completed the NDT-Tango intervention. The 26 enrolled participants per arm would yield 73% power to detect an effect of this magnitude.

### Randomization

After a participant has both qualified and agreed to participate in this trial, researchers randomize the participant into one of two groups, Tango, or HEX. A randomization schedule was created in Microsoft Excel and utilized by researchers.

### Data management

Each study participant is assigned a unique six-digit identification number that cannot be traced to their protected health information. Participant data, coded using these identification numbers, are stored in a central database using REDCap. REDCap is a secure web-based platform that is designed for clinical trials which meets both HIPAA and 21 CFR. Clinical and biomechanical data are collected by study staff who input results into REDCap manually or via data upload. PRO data is manually entered into REDCap by study participants: Tango participants fill out questionnaires within REDCap before the start and at the end of each Tango session and HEX participants fill out questionnaires within MyCap, a mobile device application of REDCap, before the start and at the end of each home exercise session.

### Statistical Analysis

All outcome analyses will be performed on an intent-to-treat basis, and all hypothesis tests will be two-sided and at the 5% significance level. Linear mixed models will be used to model changes in postural, biomechanical, and PROs. Fixed effects will be included for time, treatment, treatment-by-time interactions, and baseline outcome measure. Random effects for subjects will be used to account for correlations between observations from the same subject. The primary estimand will be the contrast representing the difference in mean change in outcome measure from baseline to week 8 between treatment groups. While we expect balance between groups due to randomization, we will adjust for potential confounders including age, body mass index, concurrent medications, and other variables if imbalance between groups occurs by chance. Pearson’s *R* will be calculated to correlate postural control and balance outcomes and PROs.

#### Visit Effect (repeated baseline testing and intervention effects calculated separately)

Per participant, postural control data (QEC) are collected on 3–5 different days prior to intervention. Descriptive data are calculated. Log-transformed data are assessed regarding the effect of visit number. Results are presented below for the subset of participants who had completed repeated baseline testing at the time of this manuscript preparation (n = 23).

##### Within-Session Effect:

At the beginning and end of each in person intervention session we collect a subset of biomechanical and PRO data with which to evaluate the effect of session. This subset includes the following biomechanical data, collected at beginning and end of sessions unless otherwise indicated: postural control variables while standing in silence (QEC) and listening to music (QECm). This protocol also includes the following EMA of PROs, collected at the beginning and end of sessions unless otherwise indicated: CIPN-20 since we last saw you (beginning only) or during the activity (end only); CIPN-20 momentary designed to assess the moment of instrument completion, brief pain inventory (BPI) since we last saw you (beginning only); BPI “right now”; brief fatigue inventory (BFI) since we last saw you (beginning only); BFI “right now”; sports concussion assessment tool version 5 (SCAT) as a generalized measure that covers neurocognitive physical and emotional symptoms impacted by neurotrauma; rating of perceived exertion (RPE) (end only); rating of perceived mental exertion (RPME) (end only); and satisfaction with intervention (end only). Each measurement is collected using a computer, a touch screen tablet, or a cell phone.

#### Intervention Effect

A linear mixed model will be applied to analyze the intervention effects for biomechanical outcomes (postural control in silence and to music, gait stability, and co-contraction index (CCI)); clinical outcomes (TUG, TUGCog, MiniBEST, 6-minute walk test (6mwt)); and PROs.

### Adverse Event Monitoring and Reporting

Both Tango and HEX participants answer a series of questions twice a week. Participants are asked to report adverse events at the beginning of each Tango or HEX session via REDCap or MyCap, respectively. Researchers review REDCap responses prior to the start of Tango lessons and if an adverse event is reported the PI is notified. Researchers review MyCap responses once a week and if an adverse event is reported the PI is notified.

#### Serious Adverse Events

All serious adverse events are immediately reported to the research team and PI, and then reported to the IRB, sponsor, and Safety Officer (SO) within 24 hours.

#### Nonserious Adverse Events

Any nonserious adverse events are reported to the SO in biannual meetings and are subject to review prior to receiving authorization for continuation of the research.

#### Biannual Reports

A biannual open report summarizing study progress and safety monitoring data is reviewed by the SO and representatives from the NIH/NIA. Approval of the SO is required for the trial to continue.

#### Post-trial care

Both Tango and HEX participants continue to report adverse events and CIPN-20 momentary for 6 months post-trial. Researchers monitor responses and check in with participants as needed. Upon completion of the 6 month follow up period researchers communicate their appreciation for participation; HEX participants are invited to participate in up to 16 Tango sessions and Tango participants are offered access to HEX materials and offered one instruction session for the purpose of teaching safe performance of the HEX protocol.

### Protocol Amendments

Any changes to the protocol require written amendments that must be approved by the NIH. If the PI determines that a protocol deviation is necessary for safety reasons, scheduling, recruitment, or personal accommodations for participants, the IRB will be notified immediately.

### Confidentiality

Any physical documentation containing protected health information is stored in a locked cabinet located within research or clinical designated space that is locked and/or monitored when not occupied. Digital documentation is stored in REDCap and/or on secure servers requiring password authentication that are behind secure firewalls.

### Access to Data

Study staff, OSU IRB, and representatives of the NIH have access to study data. Study staff are trained in HIPAA standards for privacy protection and do not refer to confidential information with anyone outside of the study team.

### Dissemination policy

The results of our research will be disseminated to: a) the scientific community; b) breast cancer survivors; c) persons with symptoms of neuropathy; and d) the public. The results of this research will be presented at scientific conferences, including the American Congress of Rehabilitation Medicine and American Society of Biomechanics. Additionally, results will be published in peer reviewed journals.

## Results

Results represent the first year of recruitment, enrollment, and baseline data collection for this trial.

### Recruitment

Our search of EMR returned an average of 230 survivors ≥ 40 years old who had received taxane-based cytotoxic chemotherapy in 3 years prior to recruitment start. An additional 1 survivor/month self-referred or was referred to us by advanced practice providers. We performed pre-screen via chart review for these 702 individuals. The reasons for failing pre-screen are listed in order of most common: (1) neuropathy reported as resolved or not experienced (n = 96 (14%)) and (2) lived too far from academic medical center (> 1 hour drive n = 15 (2%)). After chart review, 158 of these survivors passed pre-screen. We attempted contact by phone for all 158 and succeeded in contacting approximately 33%. Of those contacted, approximately 50% refused for the following reasons, listed in order of most common: (1) too busy (2) neuropathy resolved (3) already on a physical activity program for neuropathy management and (4) not interested. Twenty-six recruits volunteered for balance screening: 3 demonstrated postural control within normal limits and 23 demonstrated postural control that met our threshold for inclusion, thereby passing balance screening.

### Enrollment

All individuals who passed screening then consented to participate in the study (22 female/1 male) and enrolled (n = 23).

### Baseline characteristics

Baseline characteristics from this cohort, representing the first year of recruitment of this study, are reported in Table 3 in terms of age, years since last taxane exposure, postural control at screening, and functional testing results.

## Discussion

Despite extensive research, no pharmacologic intervention has significantly improved the neuromotor, functional, and patient-reported symptoms of chronic CIN, in concert, among breast cancer survivors. The rationale for this study stems from accumulating evidence that PA represents a non-pharmacologic avenue to treat chronic CIN. The scientific premise of this study is that NDT-Tango stands to achieve more gains that PA practice alone through combining PA with MBM and social engagement. On the basis of our preliminary data establishing safety, feasibility, and initial effect of NDT-Tango for survivors with CIN, we have designed a randomized controlled trial to compare effectiveness of an active control arm that delivers PA for CIN(23, 116) as home exercise v. an experimental arm that delivers NDT-Tango. We assess comparative effectiveness in terms of neuromotor, functional, and patient-reported outcomes relevant to BC survivorship. Our findings may lead to a safe, effective, simple, economical, non-pharmacologic intervention that improves CIN-related deficits and symptoms through activity that can be performed with a friend or loved one. Adding small doses of NDT as standard of care (SOC) for survivors with CIN is a simple, cost-effective solution that can be implemented anywhere in the world without major regulatory hurdles. Better functional recovery for survivors with CIN will lead to better short- and long-term health and wellness outcomes for these individuals. Therefore, the risks that participants in this study might incur are minor relative to the potential benefits of improving function and symptoms for BC survivors with CIN and measurable balance deficits.

### Trial status

The study has been active and open for enrollment since September 2021. Enrollment is expected to be completed in August 2023. Intervention delivery and follow up are expected to be completed by May of 2024. The clinical trial number associated with this trial is NCT05114005.

## Figures and Tables

**Figure 1:**
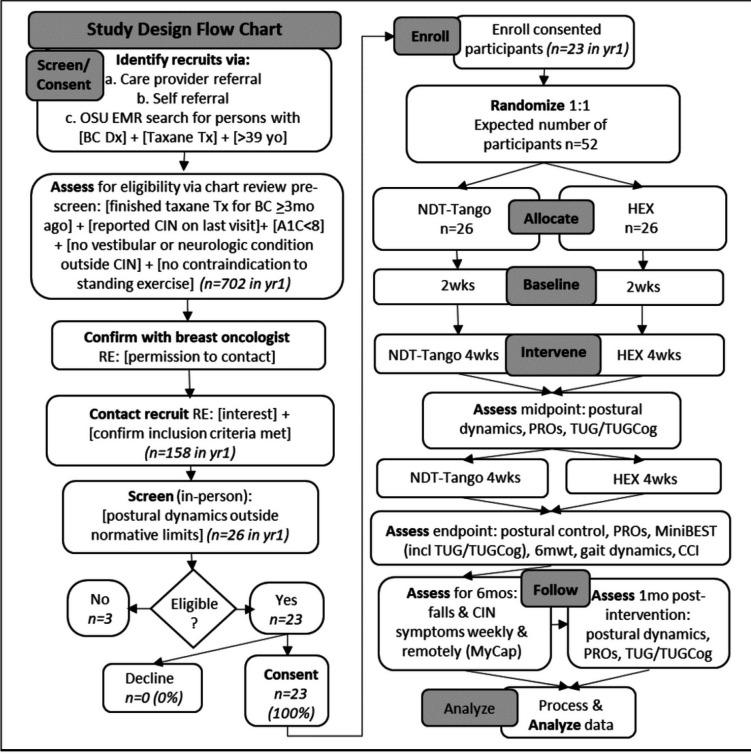
Flow chart of study design from screening through enrollment, allocation, intervention, follow-up, and analysis with assessments indicated throughout.

**Figure 2:** Legend not included with this version

**Table 1:** SPIRIT report of Intervention effect outcome measures

	Study Period						
	Enrollment	Allocation	Post-allocation				
TIMEPOINTS	3 + mo post last taxane exposure	0	Baseline (*repeated x4)	Midpoint (4wks)	Post (8wks)	1mo follow up (12 wks)	6mo follow (assessed weekly)
ENROLLMENT:							
Eligibility screen	x						
Informed consent	x						
Allocation		x					
INTERVENTIONS:							
Tango group			x	x	x	x	x
HEX group			x	x	x	x	x
ASSESSMENTS:							
Postural Control dynamics during silence (primary outcome Aim1) (QEC)			x*	x	x	x	
Postural Control dynamics during music listening (QECm)			x*	x	x	x	
TUG			x	x	x	x	
TUG-Cog			x	x	x	x	
Mini-BEST			x		x		
6mwt			x		x		
Gait dynamics (primary outcome Aim2 )			x		x		
Co-contraction index (CCI)			x		x		
Satisfaction with Intervention				x	x		
Falls incidence				x	x	x	x
Ecological Momentary Assessment (EMA) of select symptoms			x*	x	x	x	x
CIPN-20			x*	x	x	x	
BPI			x*	x	x	x	
BFI			x*	x	x	x	
Intrinsic Motivation				x	x		

**Table 2:** SPIRIT report of within-session assessments

SESSION TYPE	Baseline		NDT-Tango		HEX (Remote in MyCap app)	
TIMEPOINTS	Before beginning session (Beg)	After completing session (End)	Beg	End	Beg	End
ASSESSMENTS:						
[QEC]	x	x	x	x		
[QECm]	x	x	x	x		
[CIPN-20]	x^#^	x^^^	x^#^	x^^^	x^#^	x^^^
[BPI]	x^#^		x^#^		x^#^	
[BFI]	x^#^		x^#^		x^#^	
[CTCAE (select symptoms)]	x	x	x	x	x	x
[EMA CIPN]	x	x	x	x	x	x
[EMA Pain]	x	x	x	x	x	x
[EMA Fatigue]	x	x	x	x	x	x
[Satisfaction with session activity as neuropathy Tx]				x		x
COLLECTED TO FACILITATE TRIAL MONITORING AND INTERVENTION SHAPING:						
[Rating of Perceived Exertion (RPE)]		x		x^^^		x^^^
[Rating of Perceived Mental Exertion (RPME)]		x		x^^^		x^^^
[Intrinsic Motivation]				x^&^		x^&^

# Recall period = during the last 2 weeks OR since we last saw you (if less than 2 weeks)

^ recall period = during the activity just performed

& queried intermittently (i.e., every 2 weeks)

**Table 3:** Postural control norms among n = 23 breast cancer survivors with CIN and balance dysfunction.

Variable of interest	Age	Years since last taxane exposure	COP Ellipse Area (COPa)			COP variability in medial-lateral direction (RMSx)		COP velocity in medial-lateral direction (Vel_x)	COP Complexity
Mean	60.9	2.6	1012			5.66		11.1	0.474
SD	9.8	2.0	846			2.19		5.2	0.159
Min	40	0.25	230			1.95		4.5	0.207
Max	78	6.2	3423			11.7		23.1	0.93
Inclusion Threshold	≥ 40	≥ 0.25	> 400			> 4.0		> 11	< 0.6
% cohort who met inclusion criteria	100%	100%	75%			70%		34%	84%
*Additional variables calculated that were not used for initial screening are reported below.*									
Variable	Path length (PL)	PL Normalized	RMS_r	RMS_y	Vel_r	Vel_y	TUG(s)	TUGCog(s)	6mwt(m)
Mean	765	76.3	10.6	8.78	26.1	21.1	10.44	13.72	374.6
SD	358	33.1	4.27	3.97	11.1	9.51	2.29	4.06	67.2
Min	91	8.48	5.55	3.91	10.9	6.52	6.02	9.1	245.1
Max	1414	147	23.2	21.7	47.2	37.7	15.06	23.87	516.1

## Data Availability

The datasets used and/or established during the current study will be available from the corresponding author upon reasonable request.

## Abbreviations

BC: Breast cancer
CIN: Chemotherapy-induced neuropathy
PA: Physical activity
MBM: Music-based medicine
NDT: Neurologic Dance Training
NDT-Tango: Partnered, Adapted Argentine Tango practice
MPIs: Multiple principal investigators
min: Minutes
HEX: Home exercise
PRO(s): Patient reported outcome(s)
wk(s): Week(s)
Aim1: Dynamics of posture
Aim2: Gait
CVspeed: Coefficient of variability for stride speed
SPIRIT: Standard Protocol Items: Recommendations for Interventions Trails
Fig: Figure
mo(s): Month(s)
RPE: Rating of perceived exertion
IMI: Intrinsic Motivation Inventory
RPME: Rating of perceived mental exertion
QEC: Postural Control dynamics during silence
QECm: Postural Control dynamics during music listening
CCI: Co-contraction index
EMA: Ecological Momentary Assessment
WSV: Within-subject variability
OSU: The Ohio State University
SSCBC: Stephanie Spielman Comprehensive Breast Center
OH: Ohio
APPs: Advanced practice providers
EMR: Electronic medical records
COP: Center of pressure
partner: Invited guest
ACE: American Council on Exercise
CMES: Medical Exercise Specialist
VAS: Visual analog scale
PRO-CTCAE: Patient-Reported Outcome version of the Common Terminology Criteria for Adverse Events v5
SEI: Sample entropy, calculated using the increment method
TUG: Timed Up-and-Go test
TUGCog: Timed Up-and-Go test with cognitive dual task
MiniBEST: Mini Balance Evaluation System Test
6mwt: 6-minute walk test
MCID: Minimum clinically-important difference
IMU: Inertial measurement unit
CIPN-20: European Organization for Research and Treatment of Cancer, Chemotherapy-Induced Peripheral Neuropathy outcome measure
BPI: Brief Pain Inventory
BFI: Brief Fatigue Inventory
SCAT: Sports Concussion Assessment Tool
SO: Safety officer
COPa: COP Ellipse Area
RMSr: COP variability resultant
RMSx: COP variability in the medial-lateral direction
Vel_x: COP velocity in medial lateral direction
SOC: Standard of care
